# Effective Feature Extraction for Knee Osteoarthritis Detection on X-ray Images using Convolutional Neural Networks

**DOI:** 10.2174/0115734056360714250612080450

**Published:** 2025-06-20

**Authors:** Lei Yu, Shuai Zhang, Xueting Zhang, Heng Wang, Mengnan You, Yimin Jiang

**Affiliations:** 1 Department of School Mathematics and Computer, Wuhan Polytechnic University, Wuhan, China

**Keywords:** Deep learning, CNN, Object detection, Knee osteoarthritis, X-ray images, TPAFFKnee model

## Abstract

**Background:**

Knee osteoarthritis (KOA) is a degenerative joint disease commonly assessed using X-ray images based on the Kellgren-Lawrence (KL) criteria. Although the KL standard exists, its ambiguity often causes patients to misunderstand their condition, leading to overtreatment or delayed treatment and challenges in guiding precise surgical decisions. Moreover, the data-driven technology has been impeded by low resolution and feature distribution inconsistency of knee X-ray images. The imbalances between positive and negative samples further degrade detection accuracy.

**Objective:**

The objective of this study was to develop a deep learning-based model, namely Task-aligned Path Aggregation Feature Fusion For Knee Osteoarthritis Detection (TPAFFKnee), to improve KOA detection accuracy by addressing limitations in traditional methods. Its more accurate detection could help in terms of proper treatment for patients and precision in surgery by physicians.

**Methods:**

We proposed the TPAFFKnee model based on the EfficientNetB4 network, which introduced a path aggregation network for better feature extraction and replaced Fully Convolutional Network (FCN) with task-aligned detection as the head. Additionally, the loss function was improved by replacing the original loss function with Efficient Intersection over Union Loss (EIoU Loss) to address the imbalance between positive and negative samples.

**Results:**

The results showed that the model could accurately detect KOA categories and lesion locations based on the KL classification criteria, with a Mean Average Precision (mAP) of 93% on the Mendeley KOA dataset of 1650 knee osteoarthritis X-ray images from several hospitals. The mAP for the K2, K3, and K4 categories were 98.6%, 98.5%, and 99.6%, respectively. Compared with Faster R-CNN, SSD, RetinaNet, EfficientNetB4, and YOLOX, the proposed algorithm improved detection mAP by 14.3%, 12.4%, 15.3%, 22.7%, and 4.3%.

**Conclusion:**

This study emphasizes the importance of the EfficientNetB4 network in KOA detection. The TPAFFKnee model provides an effective solution for improving the accuracy of KOA detection and offers a promising approach for standardized KL classification in medical applications. Future research can integrate more clinical data while improving the overall landscape of healthcare delivery through data-driven automation solutions.

## 1 INTRODUCTION

KOA is a prevalent degenerative joint disease primarily affecting the knee joint, leading to progressive cartilage degradation. This degradation results in symptoms such as pain, stiffness, swelling, and reduced mobility [1]. Following the rapid advancement in medical imaging technologies, X-ray imaging has become a cornerstone in the diagnosis and assessment of KOA. It effectively reveals critical features like joint-space narrowing, subchondral bone cysts, and cartilage sclerosis, making it the predominant imaging modality for evaluating KOA due to its efficiency, cost-effectiveness, and ability to provide direct visuals of bone structures [2].

Cartilage within the knee joint facilitates smooth and frictionless movement. However, factors such as aging, obesity, prior injuries, and mechanical stress can lead to increased friction between the femur and tibia. This friction accelerates the degeneration of the articular cartilage, leading to joint-space narrowing and the formation of osteophytes (bone spurs). These pathological changes not only cause pain and stiffness but also progressively impair joint function, leading to significant mobility limitations [3]. Early-stage KOA can be managed with preventive measures, including physical activity and weight control, to slow disease progression. However, for patients with advanced KOA, the only effective treatment remains Total Knee Arthroplasty (TKA) or artificial joint replacement. Without timely intervention, KOA can severely restrict a patient's ability to walk and perform daily activities. Moreover, with the aging global population, the incidence of KOA is rising significantly, and the disease is increasingly affecting younger individuals in the 35-44 age group, marking a concerning trend [4]. Globally, patients made more than 7.5 billion visits to radiologists, underscoring the significant burden on healthcare systems and highlighting the urgent need for rapid and accurate KOA diagnosis [5]. Traditional KOA detection relies heavily on radiographic assessments performed by clinicians, a process that is both labor-intensive and subjective. Even though the KL standard exists, patients can determine their conditions for interventional therapy, easing the strain on physician resources. However, its uncertainty misleads patients in observing the lesions and accurately determining their severity, and this fails to reduce the burden on physicians. Recent advancements in deep learning and neural networks have revolutionized medical imaging, including KOA detection [6]. These algorithms are capable of recognizing complex patterns and subtle features in imaging data that may be imperceptible to the human eye, thereby enabling the extraction of nuanced differences between images. By training deep learning models on large datasets of well-annotated X-ray images, these technologies can assist clinicians, particularly those with less experience, in quickly and accurately identifying the location and severity of KOA lesions [7]. However, current deep learning research predominantly focuses on Magnetic Resonance Imaging (MRI) of KOA, with relatively few studies addressing X-ray imaging, which remains the most commonly used diagnostic tool. Furthermore, research specifically addressing the KL grading system within X-ray-based KOA detection remains limited.

The low resolution of knee X-ray images results in a lack of pixel density, making subtle features indistinguishable and poorly defined. This hampers the accurate extraction and identification of features needed for classification and localization, reducing accuracy. Consequently, the insufficient feature information and weak interaction make it challenging to recognize small bony encumbrances and minor joint space changes, leading to discrepancies and misalignment in identification, ultimately affecting the accuracy of KOA assessment. To solve the above problems, we proposed a TPAFFKnee model based on the EfficientNetB4 network. Our main innovations are as follows:

(1) To improve the feature interaction capability for the finely varied and highly subjective KOA, a modified path aggregation feature fusion network was introduced. This network increases top-down feature fusion and incorporates adaptive weights in the convolution process.

(2) A task-aligned strategy based on the layer attention mechanism was introduced in the detection header so that misalignment issues caused by the lack of alignment between the classification and localization tasks can be mitigated.

(3) To address the problem of imbalance between positive and negative samples in the training process, the loss function was improved by replacing the original loss function with EIoU Loss, using the edge length as a penalty term to align the predicted bounding box with the real bounding box. This improvement enhanced the accuracy of bounding box detection and the convergence speed of the model.

Lastly, the model was trained and validated using the KOA dataset. The results of this study enable accurate detection of KOA based on KL classification criteria and provide a theoretical basis for future developments.

The remainder of the paper is structured as follows: Section 2 describes the related work, Section 3 details the architecture of the TPAFFKnee model, Section 4 provides the results and analyses of the comparative experiments and the ablation study, and last, Section 5 summarizes the results of the research.

## 2 RELATED WORK

Early KOA detection primarily depended on doctors manually analyzing X-ray images. Those without specialized training often struggled to accurately identify lesions and assess the severity, leading to variations in diagnosis and treatment recommendations. However, with the advancement of artificial intelligence, KOA detection has become more automated. Researchers have increasingly applied deep learning techniques to KOA detection [8], focusing on improving the model architecture, optimization strategies, and loss functions, which have enhanced detection performance.

In recent years, convolutional neural networks (CNN) have significantly advanced medical imaging, particularly in the automatic detection of KOA [9]. Many studies have focused on analyzing and evaluating knee X-ray images using CNN. In 2018, Tiulpin *et al*. [10] proposed a deep Siamese CNN for KOA detection, applying it to the Osteoarthritis Initiative dataset, which included 3,000 subjects and 5,960 knee images. Their method treated the KL class as a nominal variable and used a random seed to select different knee subregions for categorical prediction. The model reduced parameters, making it more robust and less sensitive to noise, with an ROC (receiver operating characteristic) curve area of 0.93 in radiological OA diagnosis, but it had a low multi-category accuracy of 66.71%. In 2019, Wahyuningrum [11] combined preprocessing and CNN for feature extraction and used Long Short-Term Memory (LSTM) for classification, achieving an average accuracy of 75.28% and a cross-entropy loss of 0.09. In 2021, another study [12] proposed a neural network-based method to predict KOA progression using 9,280 knee MRI images from 3,268 patients. Despite introducing a neural network-based attention map, the study reported a relatively low detection accuracy of 72%.

The current accuracy of CNN for KOA detection is still insufficient and requires further improvement. In 2016, J. Antony *et al*. [13] used SVM and Sobel edge detection to localize the knee joint region and applied the VGG-16 model with transfer learning to classify KOA X-ray images based on the KL criterion, achieving a classification accuracy of 59.6%. This demonstrated that fine-tuning deep CNN models can significantly enhance classification accuracy. In 2018, Pedoia *et al*. [14] employed T2 Mapping data from 4,328 cases to segment cartilage automatically and performed voxel-wise T2 value analysis using a deep learning model to assess radiographic KOA. The model achieved an AUC of 0.82, surpassing the shallow feature extraction model's AUC of 0.78, indicating superior accuracy in radiologic diagnosis. In 2020, Nguyen *et al*. [15] introduced an extension based on the Siamese network and a semi-supervised learning technique to address limited data volume. By focusing on medial and lateral knee joint regions and generating samples near the data manifold, the Semixup model improved accuracy by 5.7% over the baseline, effectively assessing KOA with minimal labeled data. In 2022, Huo *et al*. [16] evaluated knee cartilage defects using deep learning, incorporating an attention mechanism into the loss function to focus on the cartilage region and improve classification accuracy. Additionally, they introduced a dual-consistency loss function to enhance both label consistency and label-free consistency, significantly improving the classification and localization of cartilage defects. In 2023, Tariq *et al*. [17] improved model performance by using transfer learning to fine-tune ResNet-34, VGG-19, DenseNet 121, and DenseNet 161, integrating them into a single model. This approach eliminated differences between left and right knees, enhancing robustness. A customized ordinal loss function was also employed for ordinal classification, considering the KL levels' ordering. Furthermore, in 2023, Dharmani *et al*. [18] noted that radiograph-based OA severity detection typically achieves less than 80% accuracy and is less effective for the Indian knee osteoarthritis database. They proposed an automated algorithm for OA severity detection using EfficientNetB1 transfer learning, achieving a classification accuracy close to 89%, surpassing recent results from the OAI and MOST databases.

In summary, while MRI excels in capturing detailed changes in soft tissues, X-ray remains the preferred tool for KOA detection due to its low cost, high efficiency, and accessibility in resource-limited areas. However, previous studies have shown that X-ray-based KOA detection, particularly with the Mendeley dataset, has struggled with accuracy. To address this, we proposed the TPAFFKnee model, designed to enhance KOA detection accuracy by dynamically aligning classification and localization tasks.

## 3 MATERIALS AND METHODS

### 3.1 EfficientNetB4 Network Model

In object detection, increasing the depth, width, and resolution of a model typically enhances its performance. However, this often leads to reduced generalizability and diminished accuracy on unseen data as the model becomes more complex [19]. Traditional data enhancement techniques can also compromise the integrity of image details, further lowering model accuracy [20]. To address these challenges, we employed the EfficientNetB4 network, which utilizes the Compound Scaling Method to balance the dimensions of depth, width, and resolution, maximizing computational efficiency while maintaining high performance [21]. Furthermore, it performs well in image classification tasks with relatively low computational cost. Unlike other classical models such as Faster R-CNN, SSD, and YOLOX, EfficientNetB4 offers a good balance between accuracy and efficiency. The model's lightweight architecture makes it particularly suitable for medical image analysis, where high precision is crucial and computational resources are often limited.

EfficientNetB4, derived from EfficientNetB0, features a resolution of 380x380, a depth scaling factor of 1.8, and a width scaling factor of 1.4. As shown in Table **1**, this architecture involves nine phases. The first phase begins with a convolutional layer that extracts features from input images and reduces spatial dimensionality. The subsequent phases employ the MBConv (mobile inverted bottleneck convolution) structure for deeper feature extraction, and the final stage includes a 1x1 convolutional layer, average pooling, and a fully connected layer. This carefully scaled structure enables EfficientNetB4 to effectively extract features from knee joint X-ray images, preparing them for the subsequent feature fusion process in the neck network.

In the EfficientNet backbone network, the central module is the MBConv structure, which incorporates several key operations to optimize feature extraction. As shown in Fig. (**1**), the MBConv module begins with a 1x1 convolution, followed by Batch Normalization (BN) and the Swish activation function to upscale the input features. This is followed by a depthwise convolution (k*k) that independently processes each channel to capture spatial features. The SE (Squeeze-and-Excitation) attention mechanism is then applied to reweight the importance of each channel, enhancing the relevant features while suppressing the less important ones [22].

After the SE module, another 1x1 convolution is utilized for dimensionality reduction, followed by a Dropout layer to prevent overfitting. The final output is added back to the original input, creating a residual connection that helps stabilize the training process and improve model performance. This carefully designed structure allows EfficientNet to balance efficiency and accuracy in feature extraction, making it particularly effective for processing knee joint X-ray images in the detection of KOA.

In traditional convolutional operations, all input channels must be processed, with each point in the feature map being multiplied by the corresponding convolution kernel. As the size and number of channels increase, this leads to higher computational costs and increased model complexity, prolonging training and inference times. Depthwise Separable Convolution addresses these issues by splitting the traditional convolution process into two steps: Depthwise Convolution and Pointwise Convolution, which significantly reduces computational resource consumption and the number of parameters, thus enhancing network efficiency [23].

As illustrated in Fig. (**2**), Depthwise Separable Convolution begins by applying a depthwise convolution to a feature map with multiple channels, where each channel is convolved independently using its respective convolution kernel. This process generates a new feature map for each channel, retaining the same dimensions as the input. Since there is no interaction between channels during this step, model complexity is reduced, leading to improved efficiency. Following this, a pointwise convolution using a 1x1 convolution kernel is applied across all channels, integrating and extracting information between them to enhance feature representation capability.

The SE attention mechanism, introduced by Hu *et al*. in 2018, enhances model performance by selectively calibrating the feature responses of each channel within the network [24]. The key concept of SE is to model the interdependencies between channels, allowing the network to focus more on the most relevant features.

As illustrated in Fig. (**3**), the SE mechanism operates through two primary steps: squeeze and excitation. Initially, the input feature map, with dimensions H × W × C, undergoes a global average pooling operation during the squeeze phase, reducing it to a 1 × 1 × C feature map. This step compresses spatial information, concentrating on channel-specific features. In the excitation phase, the compressed features pass through a fully connected (FC) layer, where the number of channels is first reduced using a ReLU activation function and then restored using a sigmoid function. This process generates excitation values between 0 and 1 for each channel, which are used to re-scale the original feature map. By multiplying the original feature map by these excitation values, the SE mechanism dynamically emphasizes important features while suppressing less significant ones, thereby enhancing the model's capacity to capture critical information.

### 3.2 EfficientNetB4 Model Improvement Strategy

#### 3.2.1 Path Aggregation Feature Fusion

The Feature Pyramid Network (FPN) in the EfficientNetB4 backbone leverages lower-level feature maps for precise localization and higher-level maps for semantic richness [25]. However, traditional FPNs have limitations in effectively enhancing deeper feature maps, leading to suboptimal feature fusion [26].

To address these limitations, the Path Aggregation Network (PAN) was integrated into the FPN structure, resulting in the Path Aggregation Feature Pyramid Network (PAFPN), as illustrated in Fig. (**4**). The PAFPN takes the three effective feature layers (P1, P2, and P3) extracted by the EfficientNetB4 backbone. Each of these layers is standardized to 256 channels through a convolution operation, allowing feature maps with different resolutions to be effectively merged. The P1 feature map undergoes 2x up-sampling and is combined with higher-level features, resulting in the refined feature maps f2 and f1, which provide enhanced resolution and richer feature representation.

In this network, we further refine these fused feature maps through additional convolution operations with a 3x3 kernel, a stride of 2, and a padding of 1. This process, followed by ReLU activation, not only preserves spatial accuracy but also injects detailed information into deeper layers, which is critical for effective feature extraction. The bottom-up enhancement in this structure ensures that precise localization signals are maintained across all levels, significantly improving the overall efficiency and effectiveness of the feature pyramid.

By integrating PAN with FPN, the PAFPN structure enhances the model's ability to learn complex features, leading to improved detection accuracy and faster convergence during training.

#### 3.2.2 Task-Aligned Strategy

Traditional classification detection networks often face challenges due to the different learning mechanisms for classification and localization, resulting in varied spatial distributions of features and misalignment when using independent branches for prediction [27]. The lack of interaction between classification and localization scores leads to inconsistent predictions. Additionally, anchorless detectors typically select anchors based on geometric proximity to the object's center, while anchor-based detectors rely on calculating the Intersection over Union (IoU) between the anchor box and the true label. However, the optimal anchor points for each task often differ depending on the object's shape and features. Widely used sample allocation schemes are task agnostic, making it difficult to achieve consistent predictions for both tasks. To address these limitations, the introduction of a task-aligned mechanism in the detection head further aligns the two tasks in the prediction while preserving the kneecap properties, improving the interaction between model classification and localization [28].

As shown in Fig. (**5**), the fused feature maps from the neck are first processed by a simple feature extractor. These extracted features are then fed into the Task-aligned Predictor (TAP) for classification and prediction. The feature extractor enhances the interaction between classification and localization by learning task-interacting features across multiple convolutional layers. This approach provides multilevel features with effective receptive fields at various scales for both tasks. The feature extractor uses N consecutive convolutional layers and activation functions to compute task interaction features, as represented in Equation (**1**), where conv_k_ and *δ* denote the kth convolutional layer and ReLU function, respectively.

**Table:** 

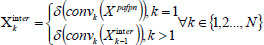	(1)

The computed interaction features are fed into two TAPs for classification and localization. As shown in Fig. (**6**), TAPs can better understand each other's states while performing these tasks on the interaction features. However, the single-branch design can cause feature conflicts, as classification and regression tasks have different goals and focus on different receptive field levels. To address this, a hierarchical attention mechanism is introduced to dynamically compute task-specific features at various levels, promoting task decomposition. These task-specific features are calculated separately for classification and localization, as shown in Equation (**2**).

**Table:** 

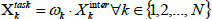	(2)

Where *ω*k is the kth element learned from layer attention *ω*ϵRN, and *ω* is computed from cross-layer task interaction features and captures dependencies between layers, computed as shown in Equation (**3**):

**Table:** 

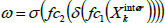	(3)

Where fc_1_ and fc_2_ are two fully connected layers. σ is a sigmoid function and X^inter^ is obtained by applying average pooling X^inter^, which is the connectivity feature of the interval X_k_^inter^. The average pooling function is a σ. Finally, the result of classification or localization is predicted from each *X^task^*, as shown in Equation (**4**):

**Table:** 

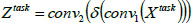	(4)

Where *X^task^* is the connected features of *X^task^* and *conv*1 is a 1×1 convolutional layer used for dimensionality reduction. The sigmoid function is then used to convert the *Z^task^* task to a dense classification score PϵR^H×W×80^ or an object bounding box *B*ϵ*R*^H×W×80^ obtained by distance-to-bound box conversion.

In the prediction step, the classification prediction features are adjusted by modifying the spatial distribution of the two predictions: p (probability) and b (bounding box). This is achieved by adjusting the classification prediction features based on the categorization features or the localization features. The predictions in the two tasks are aligned using the computed task-interaction features, and the alignment method is executed separately for the two tasks. The classification prediction goes by adjusting the classification prediction through the spatial probability map *M* ϵ *R*^*H*×*W*×1^, which is shown in Equation (**5**):

**Table:** 

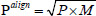	(5)

where M is computed from the interaction features, allowing it to learn a certain degree of consistency between the two tasks at each spatial location. Simultaneously, spatial offset mapping *O* ϵ *R*^*H*×*W*×8^ is learned from the interaction features for aligning the predicted bounding box at each location. Specifically, the learned spatial offsets enable the most aligned anchor point to recognize the best boundary prediction around it, and the bounding box prediction is shown in Equation (**6**):

**Table:** 

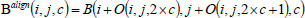	(6)

where the index (i, j, c) denotes the (i, j) spatial location at channel c in the tensor. Equation (4.6) is implemented using bilinear interpolation, which has negligible computational overhead due to the small size of the channels of B. The offsets for each channel are learned independently, which means that each boundary of the object has its own learned offset, allowing for more accurate predictions for all four boundaries since each boundary can be learned separately from the most accurate anchor point in its neighborhood. The alignment mappings M and O are automatically learned from the interaction feature stack, where *conv*_1_ and *conv*_3_ are two 1 × 1 degenerate convolutional layers, and M and O are computed as shown in Equations (**7** and **8**):

**Table:** 

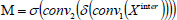	(7)

**Table:** 

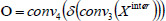	(8)

#### 3.2.3 Loss function improvement

The loss function for object detection typically includes two components: classification loss and regression loss. In the EfficientNetB4 detection network, the L1 loss function is used, but its gradient near zero is not constant, which can cause excessive updates as the model nears the optimal solution, leading to fluctuations in the loss. This issue may hinder fine-tuning in later training stages, particularly when predicted values are close to the true values. Additionally, when calculating the bounding box (bbox) loss, the independent derivation and summation of the four points do not align with actual IoU interactions. To address this, the EIoU Loss is introduced, which considers the aspect ratio while correlating the four factors x, y, w, and h, thereby increasing the model detection effectiveness.

The development of EIoU Loss follows a progression from IoU Loss to GIoU Loss, DIoU Loss, CIoU Loss, and at last, EIoU Loss. IoU Loss focuses on the area of overlap between the detection and target frames. GIoU Loss extends this by addressing cases where the bounding boxes do not overlap. DIoU Loss incorporates the distance between the centroids of the bounding boxes, while CIoU Loss further refines the model by considering overlap, centroid distance, and aspect ratio. EIoU Loss builds on CIoU Loss by introducing a penalty function that directly penalizes prediction errors. In CIoU Loss, the relative ratio of width and height is used rather than their absolute values. However, under certain conditions, the penalty term based on this ratio in CIoU Loss may fail. The calculation in CIoU Loss is represented as:

**Table:** 

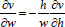	(9)

From equation (**9**), it can be observed that the gradient values *∂v*\*∂w* and *∂v*\*∂h* of *w* and *h* have opposite signs, and when one of the values of w and h increases, the other must decrease, and the two increasing and decreasing states cannot remain the same.

As shown in Fig. (**7**), the blue color represents the ground truth, while the pink, red, and green colors represent the predicted values of the three loss functions. In the figure, GIoU uses the area of the smallest closed region containing two bounding boxes minus the area of the union set as the penalty term, which can cause GIoU to expand the union set before optimizing the IoU. CIoU, on the other hand, struggles with simultaneous adjustments in width and height. For instance, in the second row of Fig. (**7**), the anchor box is larger than the object to be detected, yet the prediction box width continues to expand during optimization. In contrast, EIoU offers faster convergence. The EIoU Loss is represented by Equation (**10**):

**Table:** 

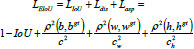	(10)

From equation (10), it can be concluded that *EIoU_LOSS_* is composed of three parts: the IoU loss *L_IoU_*, the distance loss *L_dis_*, and the edge length loss *L_asp_*. The IoU is the ratio between the predicted and true bounding boxes. Where IoU denotes the intersection and concurrency ratio of the predicted and real bounding boxes, ρ denotes the Euclidean distance function, b and *b^gt^* denote the predicted and real centroids, *w* and w^gt^ denote the predicted and real widths, *h* and *h^gt^* denote the predicted and real heights, *c_w_* and c_h_ are the normalization coefficients used to normalize the widths and heights. EIoU directly uses the edge lengths as the penalty term, and the predicted bounding boxes are better aligned with the true bounding box and, hence, have faster convergence in terms of localization accuracy.

### 3.3 TPAFFKnee Model Architecture

Based on the improvements to EfficientNetB4, we proposed the TPAFFKnee model. The structure of TPAFFKnee is shown in Fig. (**8**). The input image first passes through the EfficientNetB4, which extracts features from the fourth, fifth, and sixth layers, each containing different spatial resolutions and levels of semantic information. These features are then fed into the improved Neck network for fusion. By enhancing the bottom-up feature fusion mechanism, richer target textures and positional information from the shallow layers are effectively transferred to the deeper layers of the feature map.

The TPAFFKnee head consists of two sub-networks that interact through the Task-aligned Learning (TAL) strategy- the location regression sub-network and the classification prediction sub-network. The feature maps from the neck first pass through six convolutional layers with a 3x3 kernel size and a step size of 1. The input of 256 channels is divided into 32 groups for group normalization, with ReLU as the activation function. These features are then passed to the two TAPs, which include a hierarchical attention mechanism to

Capture dependencies between layers by dynamically computing task-specific features, enabling cross-layer interactions. TAP adjusts classification predictions using spatial probability maps and aligns the tasks by learning spatial offset maps to refine the classification prediction bounding box. The features processed by TAP are dynamically assigned for classification and regression, with the final outputs interactively refined through the TAL mechanism to enhance detection efficiency.

The loss function of TPAFFKnee in the classification task uses Focal Loss to mitigate the imbalance between negative and positive samples during the training process. The design of this loss function helps mitigate class imbalance by down-weighting the impact of negative samples (which dominate in most medical datasets) and up-weighting the impact of positive samples, which are often harder to detect. By focusing on the minority class, the model becomes more sensitive to positive samples, improving the detection performance for osteoarthritis lesions while reducing the impact of abundant negative samples. For the regression position loss, it uses EIoU Loss. Thus, the total loss function of TPAFFKnee can be expressed by Equation (**11**).

**Table:** 

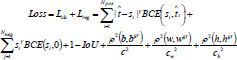	(11)

Where i denotes the i-th anchor point among the Npos positive anchors corresponding to an instance, j denotes the j-th anchor point among the Nneg negative anchors, γ is the focusing parameter, and *b_i_* and *b_i_* denote the predicted bounding box and the corresponding real bounding box, respectively. t is a threshold parameter used to modify the loss function by adjusting the importance of the samples that are difficult to categorize. In the context of our model, t is defined as: if the sample is positive, t = 1; if the sample is negative, t = 0. *s* is a scale factor that is used to modify the impact of the loss function based on the sample difficulty. In the case of imbalanced data, we introduce s to give more weight to hard examples (typically the positive samples) that are often misclassified. The scale factor is defined as in Equation (**12**).

**Table:** 

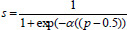	(12)

whereis *α* hyperparameter that controls the sensitivity of the weighting, and p is the predicted probability of the positive class. s increases the weight for positive samples that are difficult to classify and reduces the weight for easier samples.

**Table:** 

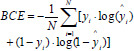	(13)

In Equation (**13**), N is the total number of pixels in the image. *y_i_* is the true label of the i th pixel (0 or 1). *ŷ_i_* is the probability that the ith pixel belongs to the foreground as predicted by the model (a value between 0 and 1). The standard BCE loss can be modified by incorporating a weighting factor (like s) to focus more on hard-to-classify examples.

The specific implementation steps of the proposed model in this paper are summarized in Table **2**.

## 4 RESULTS AND ANALYSIS

### 4.1 Experimental Data Sets

This paper utilized the Mendeley dataset IV, comprising 1650 knee osteoarthritis X-ray images from several hospitals, acquired using a PROTEC PRS 500E X-ray machine. The dataset includes 514 images in the normal category (grade 0), 477 suspicious (grade 1), 232 mild (grade 2), 221 moderate (grade 3), and 206 severe (grade 4). The original images were 8-bit grayscale with a resolution of 162 × 300. Orthopedic sur-geons from a tertiary care hospital guided the annotation pro-cess and the dataset was formatted according to the VOC data-set standard. Semantic labels and bounding box coordinates were manually annotated using LabelImg, and all image information was stored in CSV files. The model was tasked with classifying the KOA grade and detecting the lesion location. To enhance classification accuracy and generalization, images were augmented by horizontal flipping, rotation, and random cropping during runtime. The dataset was divided into training and test sets in an 8:2 ratio, as detailed in Table **3**.

Transfer learning is a machine learning strategy that transfers knowledge from a source task to a related target task [29]. This approach leverages existing knowledge, reducing the need for large datasets and shortening learning time. Typically, it involves using pre-trained models on large datasets and fine-tuning them for specific applications [30]. In this study, to enhance knee osteoarthritis detection accuracy and save training time, we used model parameters pre-trained on the ImageNet dataset instead of training from scratch. This strategy significantly shortened the training cycle, accelerated convergence, and proved highly efficient in data-limited and resource-constrained scenarios, effectively balancing computational cost and performance.

### 4.2 Experimental Environment

All experiments in this paper were conducted on a computer with Windows 11 as the operating system, a powerful processor 4.10GHz Intel(R) Core(TM) i7-11800H suitable for handling complex computational tasks, and a GPU of NVIDIA GeForce RTX 3060 configuration.

To adapt to the current hardware devices, the TPAFFKnee model in this paper used AdamW, a variant of the Adam-based optimizer, for model optimization. The initial learning rate was set to 0.0002 with a weight decay coefficient of 0.0001. To ensure the normalization effect of the network, there was no decay in the weights of the normalization layer. A stepwise learning rate was used, shrinking the learning rate by a factor of 10 at the 8th and 11th epochs. Additionally, a linear warm-up strategy for the learning rate was introduced, with the number of iterations for a warm-up set to 500 and the warm-up ratio set to 0.001. The model was trained for a total of 50 epochs with a batch size of 1.

### 4.3 Evaluation Indicators

In the field of object detection, several evaluation metrics are commonly used to assess the performance of the model. These metrics include the Average Precision (AP), Mean Average Precision (mAP), Precision-Recall (PR) curve, and detection speed.

Since the non-objects in the image are considered background, the prediction boxes are categorized as correct or incorrect. Thus, four different boxes are generated at the time of judgment:

Precision (P) refers to the ratio of the number of correct predictions to all detection results, as shown in equation (**14**):

**Table:** 

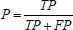	(14)

Recall (R) refers to the ratio of the number of correct predictions to all true results, and it is shown in equation (**15**):

**Table:** 

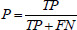	(15)

True Positive (TP): The prediction box is a correct prediction, and the IoU with the true value box is greater than the threshold.

False Positive (FP): The background in the picture is incorrectly recognized as an object.

True Negative (TN): There is a background in the picture, and the model does not form a prediction frame here.

False Negative (FN): No prediction box successfully recognizes the object, which usually occurs with small objects and occlusion.

The mAP for multiple categories is calculated by averaging the AP of each object category. Varying the threshold values will yield different detection results, affecting precision and recall rates. By adjusting the threshold to change the recall rate between 0 and 1, the precision rate will adjust accordingly, generating a set of precision-recall values that can be plotted to form a PR curve. The area under the PR curve represents the AP value, with a larger area indicating better model performance and a good balance between recall and precision. Different IoU thresholds, typically set at 0.5 or 0.75 in current object detection algorithms, will also impact the AP value. The calculation of AP is shown in Equation (**16**):

**Table:** 

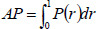	(16)

The AP value measures how well the model detects a single category of targets, but many scenarios involve the detection of multiple targets. Therefore, it is necessary to evaluate these multi-category target detection models using mAP. mAP is the main evaluation metric for object detection and is the metric used in international object detection algorithm competitions. It is derived by averaging the AP values of all categories. Its calculation is shown in Equation (**17**), where N (Class) is the number of classes.

**Table:** 

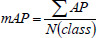	(17)

Besides accuracy metrics, model detection speed is also evaluated, represented by Frames Per Second (FPS). In object detection, FPS indicates how many images the model can process per second. For example, if the model takes 0.05 seconds to process an image, the FPS value would be 1/0.05=20. A higher FPS value means faster detection speed. The calculation of FPS is shown in Equation (**18**), where S represents the time required to detect an image:

**Table:** 

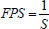	(18)

Object detection is different from image classification, as it evaluates the detection effect by judging the degree of overlap between the output bounding prediction box and the real box, usually using Intersection over Union (IoU). IoU is defined as the ratio of the intersection of two bounding boxes to their union, taking values in the range [0,1]. The larger the IoU value, the higher the degree of overlap between the two boxes. Typically, 0.5 is chosen as the IoU threshold to verify the accuracy of the prediction box. When the IoU value is greater than 0.5, the box is considered a correct prediction; otherwise, it is invalid. The calculation *IoU*_*A*,*B*_ is shown in Equation (**19**):

**Table:** 

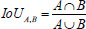	(19)

This prediction is therefore considered correct when and only when the category is judged correctly and the IoU with the truth box is greater than a threshold. Otherwise, it is an incorrect prediction. The prediction is shown in Equation (**20**), where *C_pred_* denotes the categorical category of the prediction box and *C_gt_* denotes the categorical category of the truth box.

**Table:** 

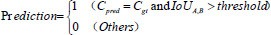	(20)

### 4.4 Ablation Study

This study analyzed the impact of various architectural components on the TPAFFKnee model's performance through ablation experiments. Ablation experiments are commonly used in object detection to validate the effectiveness of individual modules and demonstrate their combined contribution to model performance. The experiments involved four different configurations, with results shown in Table **4**. The baseline model, EfficientNetB4, achieved a mAP of 86.1% after 50 training iterations. Each subsequent experiment introduced incremental improvements.

In Case 1, we introduced PAFPN, which enhances the bottom-up feature aggregation process, allowing for more effective utilization of lower-level feature maps. This improvement led to a 2.2% increase in the model's mAP, primarily due to PAFPN's ability to capture fine-grained features of KOA lesions that were previously difficult to detect. By optimizing the fusion of features across different levels of the network, PAFPN enables better detection of subtle details in the images. This result highlights the effectiveness of bottom-up feature fusion in enhancing detection accuracy, particularly for complex medical imaging tasks like KOA lesion detection.

In Case 2, we added a task-aligned mechanism to resolve the misalignment between categorization and localization tasks. This mechanism aligns the feature extraction process more closely with the specific requirements of KOA lesion detection. By adjusting the model's attention and weighting the loss function, it prioritizes the detection of small lesions, ensuring the network focuses on the most critical regions of the image. The addition of the task alignment mechanism resulted in a further 0.6% boost in mAP, proving essential for the accurate detection of KOA lesions.

Building on Case 2, TPAFFKnee introduced additional enhancements by incorporating EIoU Loss and a penalty term to tackle the category imbalance in the dataset. EIoU Loss improves bounding box predictions by emphasizing lesion localization accuracy, while the penalty term helps mitigate the imbalance where non-osteopathic (negative) samples far outnumber osteopathic (positive) cases. These improvements led to a significant 4% increase in mAP, demonstrating that the introduction of EIoU Loss and the imbalance penalty term not only accelerated the network's convergence but also greatly enhanced both localization and detection accuracy.

Similar improvements were observed in the training dataset. Fig. (**9**) illustrates the changes in mAP values across epochs for Case 1, Case 2, and TPAFFKnee. Fig. (**9a**) shows the general trend from 0 to 50 epochs, while Fig. (**9b**) zooms in on the detailed changes from 10 to 50 epochs. From the observations in Fig. (**9b**), it is clear that TPAFFKnee (green line) consistently achieves higher mAP values, indicating that its model is better trained and more accurate. The relatively smooth curve further suggests that TPAFFKnee maintains good consistency and stability throughout the training process. This performance underscores that TPAFFKnee is more effective for KOA detection.

To evaluate the performance of the classification models in each category, we generated confusion matrices for both EfficientNetB4 and TPAFFKnee. Fig. (**10**) displays these confusion matrices for the KOA detection task, where the horizontal rows represent actual categories, and the vertical columns show predicted categories. The percentage values in each cell reflect the predicted distribution, while the diagonal values indicate correct classifications.

In Fig. (**10a**), EfficientNetB4 correctly classifies 83% of lesion-free instances as K0 and 77% of severe lesions as K4. However, 15% of lesion-free cases are misclassified as K1 (Doubtful). The model struggles with Mild (K2) and Moderate (K3) lesions, with a notable portion of K2 cases (30%) misclassified as K1. Fig. (**10b**) shows that TPAFFKnee achieves higher accuracy for K0 (82%) and K4 (75%) compared to other configurations. However, its detection rate for Mild (K2) is lower at 48%, indicating difficulty in identifying mild lesions. TPAFFKnee performs well in classifying K1, K2, and K3, with the highest accuracy in Moderate (K3) at 65%. Notably, Doubtful (K1) is often misclassified as no lesion, likely due to visual similarities. TPAFFKnee's strong performance in detecting Moderate lesions can be attributed to its task-aligned strategy with a layer-attention mechanism, which enhances feature interaction across layers and improves accuracy, especially for cases with atypical presentations.

Fig. (**11**) illustrates the performance of the TPAFFKnee model on knee arthritis datasets, validating its detection capabilities. The TPAFFKnee model effectively reduces under-detection (failing to detect arthritic lesions) and mis-detection (incorrectly labeling regions without lesions) compared to the EfficientNetB4 network. In the figure, each bounding box in the X-ray image represents the model's predicted location of arthritic lesions, with values indicating confidence or accuracy scores and different colors representing various arthritis categories. Accurate lesion localization is crucial for guiding minimally invasive surgery and assessing lesion progression, which are essential for proper diagnosis and treatment planning. The TPAFFKnee model demonstrates superior localization and detection accuracy, showing greater robustness against variability and noise within the dataset. This adaptability to blurring, occlusion, similar anatomical structures, and variations in image quality makes it highly reliable for clinical applications, enhancing the effectiveness of automated diagnostic systems.

Based on the training results, four sets of PR plots are created for IoU thresholds of 0.5 and 0.75, respectively, as shown in Fig. (**12**).

EfficientNetB4 PR curves **(a)**: At IoU=0.5, the model maintains high precision in the interval of low to medium recall, with a substantial drop in precision when recall approaches one. At IoU=0.75, the precision decreases more significantly as recall increases, reflecting decreased model performance under more stringent IoU thresholds.

Case 1 PR curve **(b)**: The precision at IoU=0.5 is slightly higher than that of EfficientNetB4, suggesting improved model performance under looser IoU thresholds after the introduction of path aggregation feature fusion. The precision curve for IoU=0.75 also shows an improvement over EfficientNetB4 at high recall intervals.

Case 2 PR curve **(c)**: At both IoU thresholds, the precision of Case 2 is higher than that of Case 1 and EfficientNetB4, indicating that the addition of the task-aligned strategy on top of the path aggregation feature fusion further improves model performance. At IoU=0.75, the precision curve is flatter than the previous models, indicating maintained performance under strict IoU thresholds.

TPAFFKnee PR Curve **(d)**: This is the best performer among the four models, especially at IoU=0.75, where precision remains high at high recall, suggesting significantly enhanced precise target detection ability. Regardless of the thresholds of 0.5 and 0.75, the area enclosed by the PR curve (*i.e*., the average precision) of TPAFFKnee is higher than the other three methods. The precision does not decrease with higher recall, illustrating the effectiveness of the task-aligned-based path aggregation feature fusion network proposed in this paper.

### 4.5 Performance Comparison

Fig. (**13**) shows the classification loss, localization loss, and total loss graphs for EfficientNetB4 and TPAFFKnee during training. Fig. (**13a**) shows the classification loss, Fig. (**13b**) the regression loss, and Fig. (**13c**) the total loss. The horizontal axis represents the number of iterations, while the vertical axis shows the loss value. The graphs indicate a significant reduction in loss after 800 iterations, attributed to interval learning rate adjustments. The models begin to converge after 3,000 iterations, with TPAFFKnee’s classification, regression, and total loss values approaching the global optimum at around 0.08, 0.25, and 0.33, respectively.

Fig. (**14**) shows the mAP values of TPAFFKnee and EfficientNetB4 during training. The horizontal axis represents the number of training epochs, and the vertical axis shows the mAP value, which indicates the performance of an object detection model-higher mAP values signify better performance. The figure demonstrates that as the number of iterations increases, the mAP values for both models rise until they stabilize.

In the initial iterations, both models quickly learn dataset features, leading to a rapid increase in mAP values. After a certain number of epochs, the mAP values reach a stable high level, with TPAFFKnee outperforming EfficientNetB4. This superior performance is due to the task-aligned strategy and the use of focal loss and EIoU loss functions, which together accelerate model convergence and maintain high performance throughout the training cycle, resulting in better stability and detection accuracy for TPAFFKnee.

Table **5** shows that the TPAFFKnee model achieves a mAP of 93.1%, surpassing the 86.1% mAP of EfficientNetB4. Notably, the AP values for K2, K3, and K4 levels exceed 98%, highlighting TPAFFKnee's superior detection accuracy. Although the AP values for K0 and K1 levels are lower, the overall mAP is significantly higher. In terms of computational efficiency, TPAFFKnee operates with 640 million FLOPs, compared to EfficientNetB4's 760 million FLOPs, indicating that TPAFFKnee not only enhances detection accuracy but also reduces computational resource consumption.

## 5 DISCUSSION OF LIMITATIONS

While we conducted relevant research on the application of mainstream models for object detection in computer vision for KOA detection and made some progress, several limitations should be considered:

(1) The data source of this paper is limited to a publicly available dataset for Indian people. Due to different living habits in each country, physical conditions can vary across populations, and there is no systematic KOA dataset for the Chinese population. It is necessary to collaborate with hospitals to establish a KOA detection model specific to the Chinese people to achieve targeted object detection.

(2) The onset of KOA is related to lifestyle habits, obesity, age, and other factors. Therefore, the next step can be to combine the test with the patient's clinical situation to improve the prediction and judgment of KOA patients, providing more accurate advice and better treatment programs.

(3) The current model is not accurate enough for the detection of KL type 2. Additionally, the model performs multiple extractions and fusions of features, resulting in high complexity. In future studies, further optimization and branch reduction are needed to reduce the computational load of the model and improve its training and detection speed. The network structure can also be optimized to enhance the KOA detection accuracy and speed.

## CONCLUSION

In this paper, the TPAFFKnee model for KOA detection was proposed to address the problems of the low resolution of knee X-ray images, inconsistent feature distribution, and the inability to recognize tiny bone recumbency or slight joint space changes due to insufficient feature information and its weak interactivity. Firstly, we compared the performance of five classical object detection models-Faster R-CNN, SSD, RetinaNet, EfficientNetB4, and YOLOX-on the Mendeley KOA dataset of 1650 knee osteoarthritis X-ray images from several hospitals, where EfficientNetB4 exhibited a higher KOA recognition rate and detection accuracy due to its scale-balanced architecture. Subsequently, we developed the TPAFFKnee model, introducing the path aggregation feature fusion network and the task-aligned strategy. The former added top-down feature fusion, allowing features with different depths to flow in both directions for more efficient utilization. Simultaneously, the latter introduced the task-aligned strategy based on the layer-attention mechanism in the detection header, which alleviated the misalignment problem due to the lack of alignment between classification and localization tasks. To improve the consistency between classification and localization, the loss function was also modified to consider the distance from the center point of the bounding box and the aspect ratio, addressing the sample imbalance problem during training. Lastly, the TPAFFKnee model was fine-tuned using transfer learning to achieve a mAP of 93%, improving 7.7 percentage points over the EfficientNet benchmark and demonstrating robustness across various KOA categories. Therefore, The TPAFFKnee model provides a promising foundation for advancing KOA detection technology and addressing its current limitations.

## Figures and Tables

**Fig. (1):**
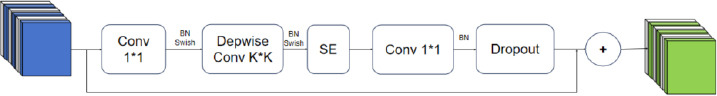
Structure of the MBConv module.

**Fig. (2):**
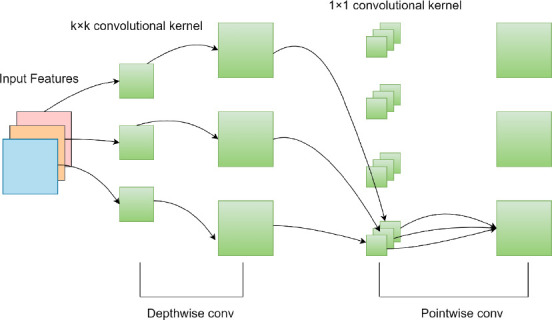
Depth separable convolutional structure.

**Fig. (3):**
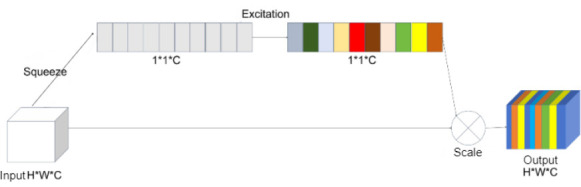
Structure of the MBConv module.

**Fig. (4):**
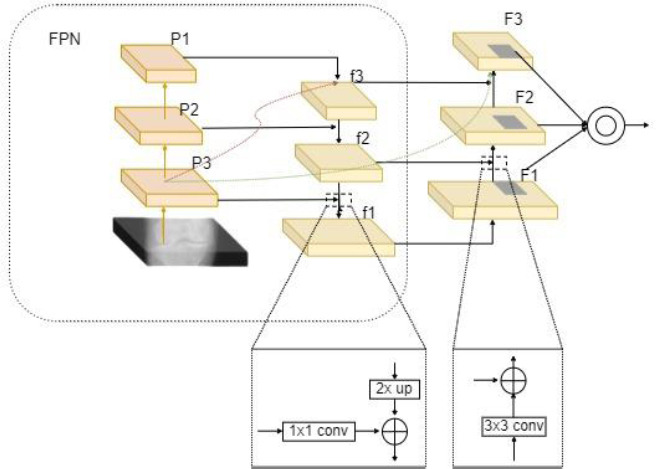
Schematic diagram of PAFPN.

**Fig. (5):**
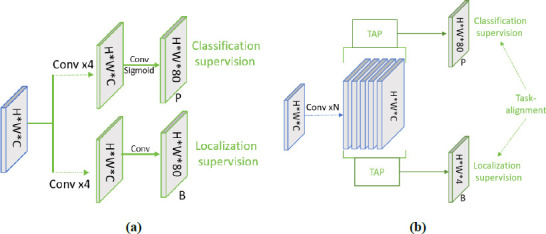
Flowchart of the head of task-aligned. **(a).** General classification and localization head. **(b).** Task-aligned mechanism head T-Head.

**Fig. (6):**
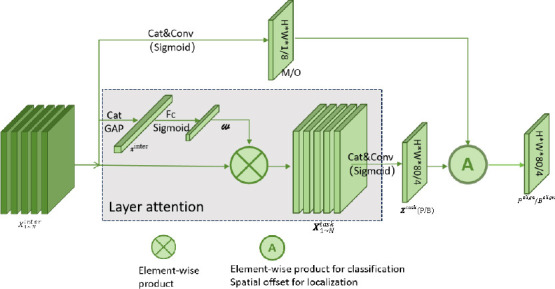
Task-aligned predictor.

**Fig. (7):**
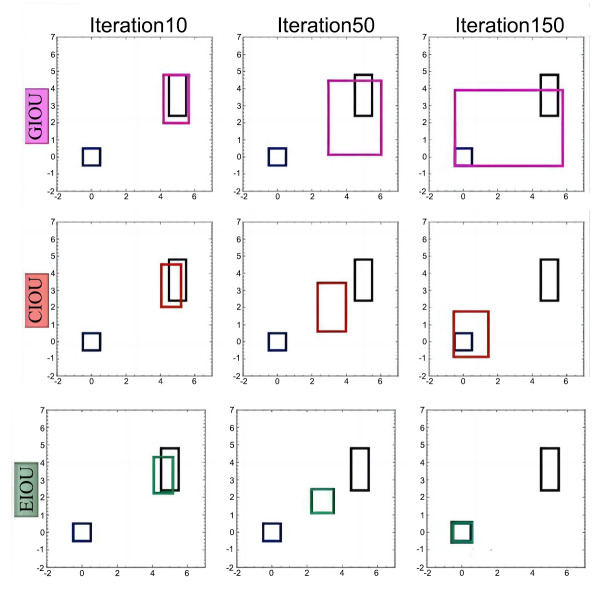
Three loss functions at the same anchor point and convergence.

**Fig. (8):**
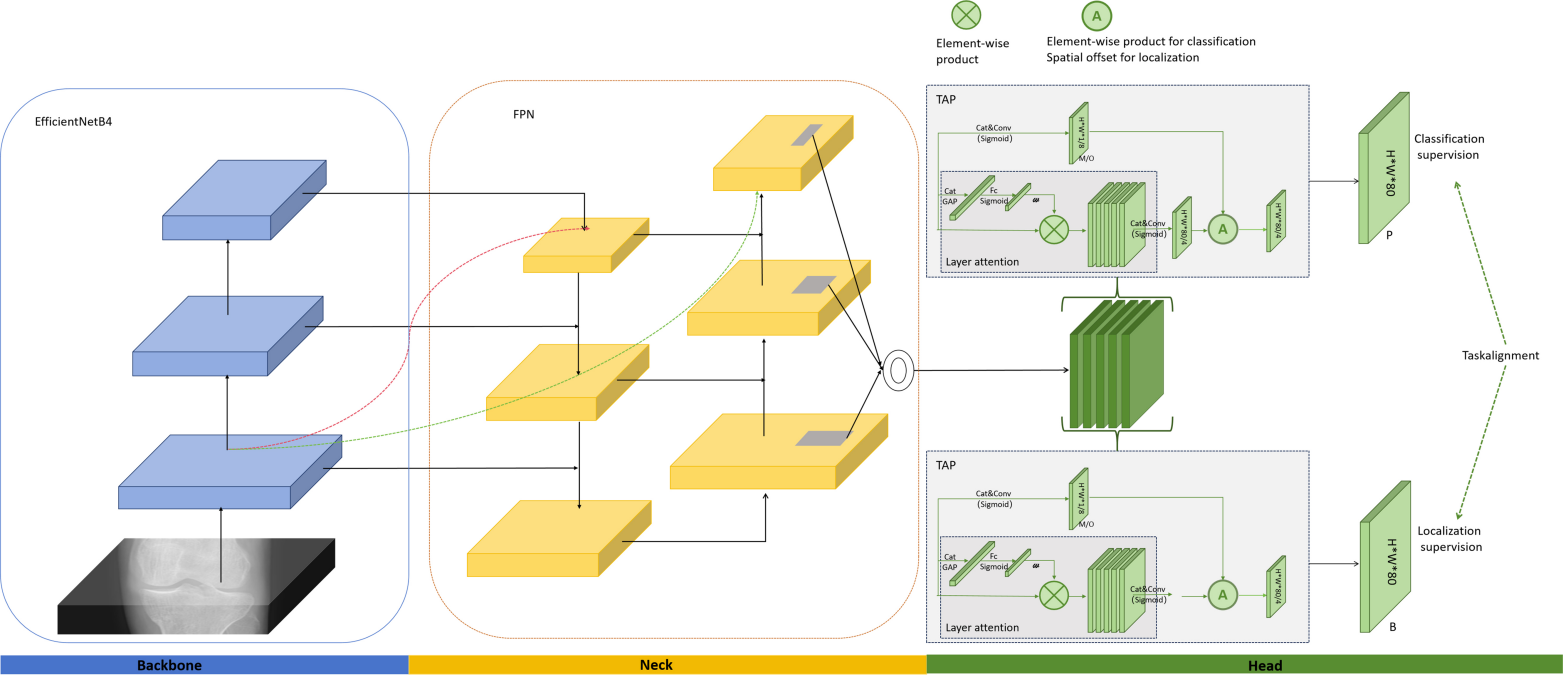
Network structure of the TPAFFKnee model.

**Fig. (9):**
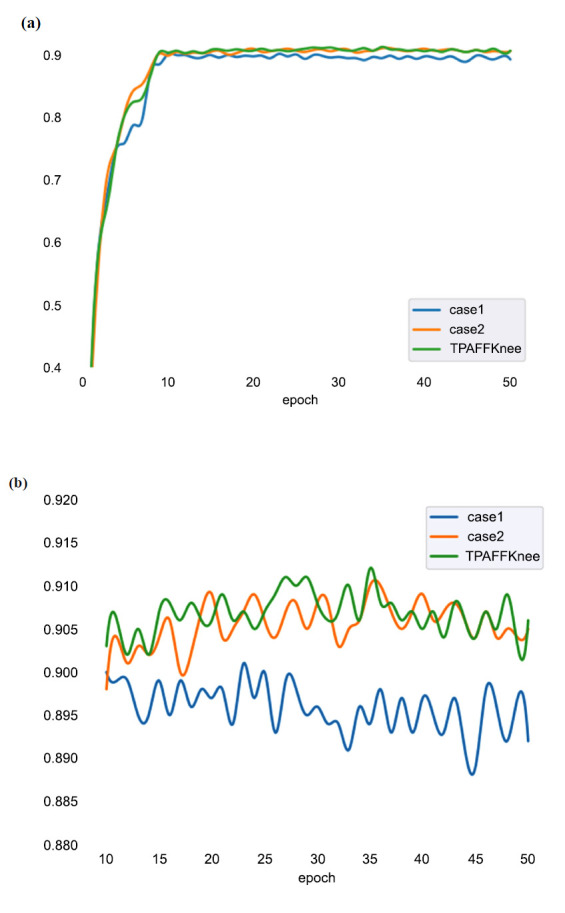
Change in training mAP values. **(a)**. Change in training mAP values from 0 to 50 epochs. **(b).** Change in training mAP values from 10 to 50 epochs.

**Fig. (10a, b):**
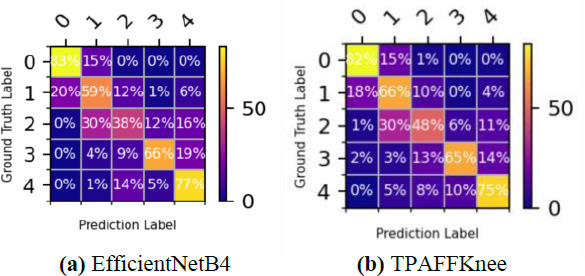
Comparison of model confusion matrices.

**Fig. (11):**
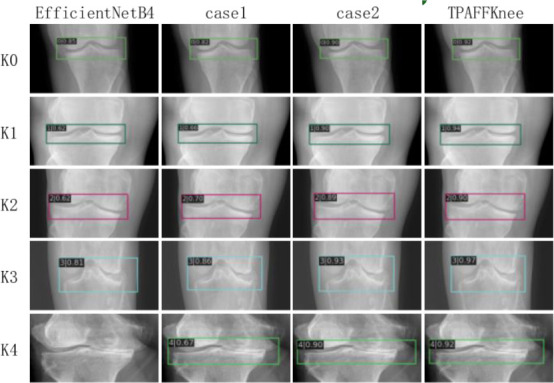
Comparison of model detection.

**Fig. (12a-d):**
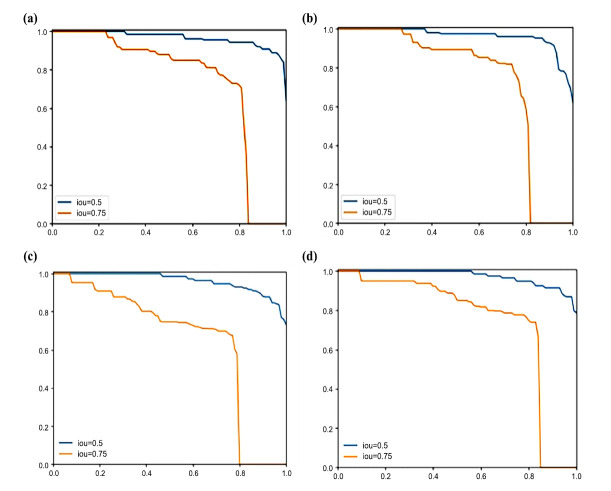
Comparison of TPAFFKnee PR at the improvement points. **(a).** EfficientNetB4 PR diagram. **(b).** Scenario 1 PR diagram. **(c).** Scenario 2 PR diagram. **(d).** TPAFFKnee PR Comparison diagram.

**Fig. (13a-c):**
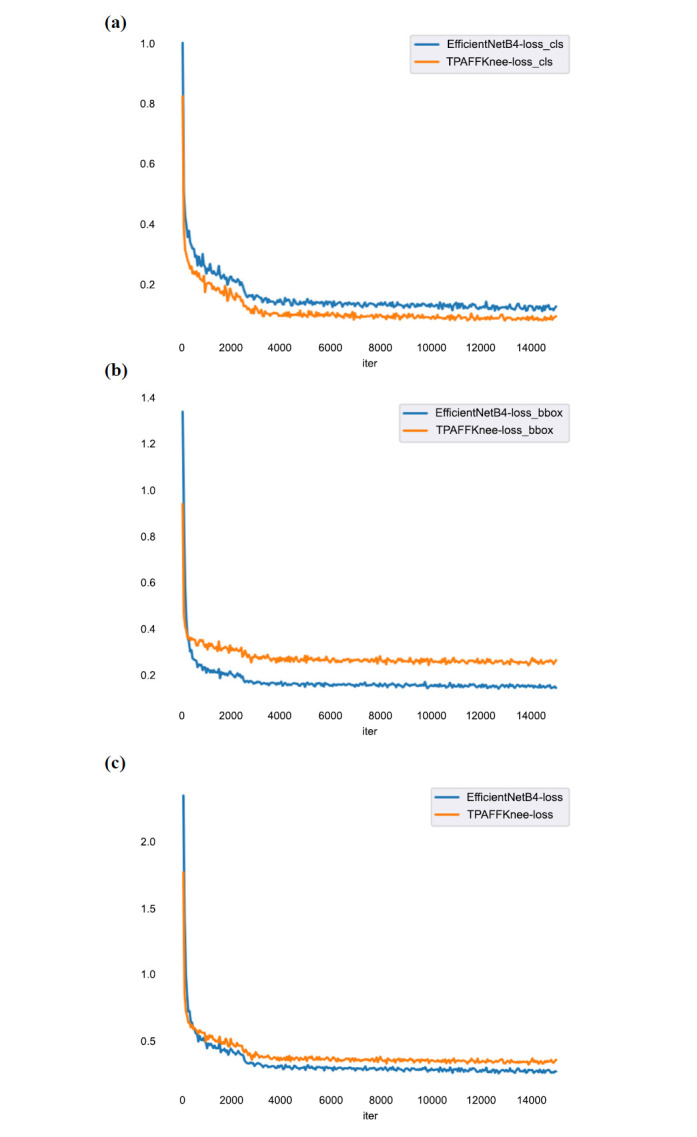
Classification loss, localization loss, and loss variation plots for EfficientNetB4 and TPAFFKnee.

**Fig. (14):**
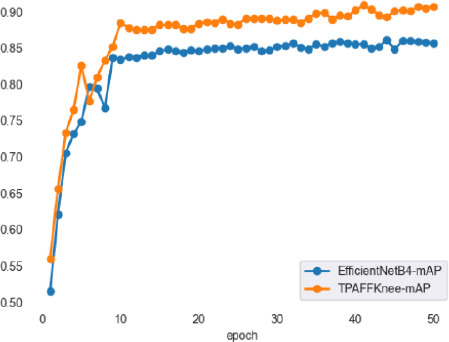
Comparison of mAP values for EfficientNetB4 and TPAFFKnee.

**Table 1:** EfficientNet-B0 network model structure.

**Stage**	**Operator**	**Resolution**	**Channels**	**Layers**
1	Conv3×3	380x380	45	2
2	MBConvl,k3×3	190x190	22	2
3	MBConv6,k3×3	190x190	34	4
4	MBConv6,k5×5	95x95	56	4
5	MBConv6,k3×3	48x48	112	5
6	MBConv6,k5×5	24x24	157	5
7	MBConv6,k5×5	24x24	269	7
8	MBConv6,k3×3	12x12	448	2
9	Conv1×1&Pooling&FC	12x12	1792	2

**Table 2:** Specific implementation steps of the TPAFFKnee model.

**Algorithm 1 The TPAFFKnee proposed in this paper.**
Input: Knee Osteoarthritis
Step 1: The model parameters are initialized using the pre-training weights of EfficientNetB4, and then the input knee joint images are sent to EfficientNetB4 to obtain C4, C5, and C6 feature maps.
Step 2: Use the path aggregation feature fusion proposed in this paper to fuse the feature maps to obtain 5 new feature maps, P3, P4, P5, P6, and P7.
Step 3: The classification branch and detection branch of TPAFFKnee with task-aligned mechanism perform detection and regression on 5 feature maps, respectively, and align the two tasks of classification and regression through the TAP prediction period and TAL mechanism.
Step 4: WHILE (end of training condition not reached) DO optimize the training of the network based on the loss of the predicted values. Then, End while.
Step 5: Test the trained model using the test dataset.
Step 6: Execute the NMS algorithm to obtain the final detection results.
Output: Output the corresponding test results.

**Table 3:** Composition and division of the osteoarthritis of the knee dataset.

**Name**	**Train**	**Test**	**All**
KL0Normal	411	103	514
KL1Doubtful	381	96	477
KL2Mild	185	47	232
KL3Moderate	176	45	221
KL4Severe	164	42	206
All	1317	333	1650

**Table 4:** Ablation study configuration.

**Model**	**PAFPN**	**TOOD**	**EIoU Loss**	**mAP**
EfficientNetB4				0.861
case1	√			0.883
case2	√	√		0.891
TPAFFKnee	√	√	√	0.931

**Table 5:** Ablation experiments results.

**Model**	**APK0**	**APK1**	**APK2**	**APK3**	**APK4**	**mAP**	**Flops**	**Params**
EfficientNetB4	0.978	0.831	0.725	0.836	0.937	0.861	7.6	25.23
TPAFFKnee	0.914	0.774	0.986	0.985	0.996	0.931	6.4	25.69

## Data Availability

The data supporting the findings of the article will be available from the corresponding author [X.Z] upon reasonable request.

## LIST OF ABBREVIATIONS

KOA: Knee Osteoarthritis
TKA: Total Knee Arthroplasty
MRI: Magnetic Resonance Imaging
CNN: Convolutional Neural Network
FCN: Fully Convolutional Network
LSTM: Long Short-Term Memory
FPN: Feature Pyramid Network
TP: True Positive
TN: True Negative
FP: False Positive
FN: False Negative
